# Improving the skills and practice of nurses to provide quality care to adolescents in conditions of vulnerability*

**DOI:** 10.1590/1518-8345.0000.3615

**Published:** 2022-10-03

**Authors:** Silvia Helena De Bortoli Cassiani, Bruna Moreno Dias, Sonja Caffe

**Affiliations:** 1 Pan American Health Organization/World Health Organization, Health System and Services Department. Washington, D.C., Estados Unidos da América.; 2 Pan American Health Organization/World Health Organization, Family, Health Promotion and Life Course Department. Washington, D.C., Estados Unidos da América.


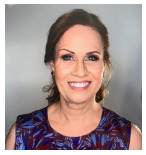



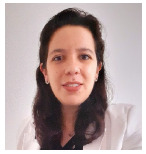



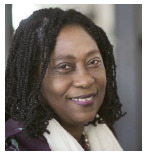


In October 2014, the Pan American Health Organization/World Health Organization (PAHO/WHO) and its Member States adopted the Strategy for Universal Access to Health and Universal Health Coverage, which defines universal access as the absence of geographical, economic, sociocultural, organizational, or gender barriers; and health coverage as the capacity of the health system to serve the needs of the population, including the availability of infrastructure, human resources, health technologies (including medicines) and financing. The Universal health strategy advocated for a robust health system, supported by motivated, well-distributed health personnel with an appropriate set of skills, to provide quality services based on the needs of the population^1^.

In September 2017, PAHO and its Member States approved the Strategy on Human Resources for Universal Access to Health and Universal Health Coverage. The lines of action of the Strategy are: strengthen and consolidate governance and leadership; to develop conditions and capacities to expand access to health and health coverage, with equity and quality; and to partner with the education sector to respond to the needs of health systems in transformation toward universal health^2^. These documents highlighted the shortage of Human Resources for Health (HRH) and its impact in the delivering of services focused on patient care and with the quality and safety required.

In September 2018, the Plan of Action for Women’s, Children’s, and Adolescents’ Health 2018-2030 was approved by PAHO/WHO Member States. The plan calls for the promotion of universal and equitable access for women, children, and adolescents to quality and comprehensive health services. Proposed actions under this objective include the development and implementation of comprehensive adolescent-responsive care, and systematic analysis and addressing of the barriers faced by adolescents in conditions of vulnerability in accessing health services.

Since 2020, the COVID-19 pandemic has impacted health services and health outcomes in the Americas, particularly in vulnerable populations and highlighted the inextricable connections between health, the economy, the environment, and social protection policies and mechanisms.

Given the impact of COVID-19 in the region, advancing, and implementing a primary health care (PHC) approach is urgently needed now more than ever. Within this context, PAHO and Global Affairs Canada initiated a project in 2021 focused on interventions to strengthen the first level of care to ensure the continuity of priority health programs, planning for the recovery of health systems over the mid-to longer term. Its actions will contribute to increasing access to and coverage of sexual and reproductive and maternal health services for women and adolescent girls by identifying and overcoming barriers to address their health needs through the provision of gender responsive, culturally sensitive, people centered, comprehensive, and quality interventions focused on the life-course approach. A sub-project is targeting the role of nurses in the improvement of health of adolescents in conditions of vulnerability.

With the collaboration of the schools of nursing of the Universidad Nacional de Colombia - Colombia, Universidad de Guayaquil - Ecuador, and Universidad Nacional Mayor de San Marcos - Perú, an analysis of *curricula* and identification of content related to adolescent health in undergraduate nursing courses was conducted. Among the results found was the need to improve the capacity of the faculty and the identification of themes related to adolescent health that need be incorporated into the training of nurses, such as behavior (decision and attitude), gender identity and sexual orientation, bullying and cyberbullying, use of digital technologies, violence in relationships, parenthood in adolescence, and pubertal delay.

Adolescence is an important stage of human development, in which adolescents, neither old children nor young adults, deal with physical, hormonal, sexual, emotional, cognitive, moral and relationship factors. Although they are generally considered a healthy group, the incidence of preventable or treatable health conditions and the barriers of access are identified as important issues affecting their health^3^.

Since 2020, this group has been affected by the direct effects of COVID-19, but also in their daily lives, with the interruption of critical health services, including mental health and sexual and reproductive health services, the disruption of education and reduced social environment and exchanges, the loss of family income, and increased exposure to violence^4^. It is critical that health care providers’ competencies and skills, and health facilities are enhanced to respond better to the needs of this age group and advance universal health coverage for adolescents^5^. Together with the lessons learned from the COVID-19 pandemic response, the global standards for quality health-care services for adolescents, published by WHO in 2015, provide clear guidance on the way forward in this respect^6^.

Given the insertion and role of nurses in health services and systems, strengthening the nursing workforce involves prioritizing policies that focus on actions in the areas of education, employment, leadership and maximizing the contributions of nurses in service delivery, as recommended in the WHO Global Strategic Directions for Nursing and Midwifery 2021-2025^7^. 

The investment in nursing professionals, in their various spheres of action and decision-making, will respond to problems such as shortages, inequitable distribution, and insufficient qualification, with a specific impact on populations in vulnerable conditions.

The Pan American Health Organization in order to overcome the need to update the knowledge of faculty and nurses in regard to adolescent health is planning several activities in the next years, such as the promotion of the course “*Salud integral de los y las adolescentes*”, available in the Virtual Campus of Public Health, and the realization of webinar series, workshops and other capacity building activities focused on the training needs identified. Additionally, analysis of policies related to adolescent health, participation of nurses in leadership positions, advocation for expanding the role of nurses in school health and primary health care and disseminating nursing practices on adolescent health will be conducted. This special issue of this journal is one of the achievements of this project.

It is expected that the articles published here will assist in the dissemination of best practices and roles of nurses in different countries, especially those countries in the project such as Bolivia, Ecuador, Colombia, Guyana, Honduras, Perú, and generate subsidies for governments, policy makers, educational institutions, professional associations, and researchers to support effective actions related to adolescent health.
